# Acute Kidney Injury Adjusted for Parenchymal Mass Reduction and Long-Term Renal Function after Partial Nephrectomy

**DOI:** 10.3390/jcm8091482

**Published:** 2019-09-18

**Authors:** Hyun-Kyu Yoon, Ho-Jin Lee, Seokha Yoo, Sun-Kyung Park, Yongsuk Kwon, Kwanghoon Jun, Chang Wook Jeong, Won Ho Kim

**Affiliations:** 1 Department of Anesthesiology and Pain Medicine, Seoul National University Hospital, Seoul National University College of Medicine, Seoul 03080, Korea; hyunkyu18@gmail.com (H.-K.Y.); zenerdiode03@gmail.com (H.-J.L.); muroki22@gmail.com (S.Y.); mayskpark@gmail.com (S.-K.P.); tonyjj88@gmail.com (K.J.); 2 Department of Urology, Seoul National University Hospital, Seoul National University College of Medicine, Seoul 03080, Korea; drboss@korea.com

**Keywords:** acute kidney injury, partial nephrectomy, parenchymal mass reduction, ischemia

## Abstract

We sought to evaluate the association of postoperative acute kidney injury (AKI) adjusted for parenchymal mass reduction with long-term renal function in patients undergoing partial nephrectomy. A total of 629 patients undergoing partial nephrectomy were reviewed. Postoperative AKI was defined by the Kidney Disease: Improving Global Outcomes (KDIGO) serum creatinine criteria, by using either the unadjusted or adjusted baseline serum creatinine level, accounting for renal parenchymal mass reduction. Estimated glomerular filtration rates (eGFRs) were followed up to 61 months (median 28 months) after surgery. The primary outcome was the functional change ratio (FCR) of eGFR calculated by the ratio of the most recent follow-up value, at least 24 months after surgery, to eGFR at 3–12 months after surgery. Multivariable linear regression analysis was performed to evaluate whether unadjusted or adjusted AKI was an independent predictor of FCR. As a sensitivity analysis, functional recovery at 3–12 months after surgery compared to the preoperative baseline was analyzed. Median parenchymal mass reduction was 11%. Unadjusted AKI occurred in 16.5% (104/625) and adjusted AKI occurred in 8.6% (54/629). AKI using adjusted baseline creatinine was significantly associated with a long-term FCR (*β* = −0.129 ± 0.026, *p* < 0.001), while unadjusted AKI was not. Adjusted AKI was also a significant predictor of functional recovery (*β* = −0.243 ± 0.106, *p* = 0.023), while unadjusted AKI was not. AKI adjusted for the parenchymal mass reduction was significantly associated with a long-term functional decline after partial nephrectomy. A creatinine increase due to remaining parenchymal ischemic injury may be important in order to predict long-term renal functional outcomes after partial nephrectomy.

## 1. Introduction

Acute kidney injury (AKI) frequently occurs after partial nephrectomy, with an incidence of up to 54% [1,2]. Furthermore, renal function gradually declines and chronic renal insufficiency may develop after partial nephrectomy [3]. A new baseline estimated glomerular filtration rate (eGFR) after early postoperative recovery following partial nephrectomy can impact survival after nephrectomy [4]. As a postoperative AKI is associated with the development of chronic kidney disease (CKD) [5], the AKI after a nephrectomy could also be associated with poor long-term renal function. However, the potential impact of an AKI after a partial nephrectomy on long-term renal function has been debated. Several studies investigated the impact of AKI after radical or partial nephrectomies, with controversy resulting [2,6,7,8]. Studies of radical nephrectomy reported that AKIs are associated with new-onset CKD [7,8], while studies of partial nephrectomy reported inconsistent results [2,6].

Furthermore, there remains uncertainty regarding how to diagnose AKI after partial nephrectomy. An AKI is diagnosed by the degree of serum creatinine elevation according to clinical criteria, such as the Kidney Disease: Improving Global Outcomes (KDIGO) criteria [9]. However, an AKI after nephrectomy is different from other surgeries, because postoperative serum creatinine elevation could be due to the parenchymal mass reduction by surgical resection as well as ischemic injury of the remaining renal nephrons [2,4,10]. Therefore, conventional criteria which do not consider the parenchymal mass reduction by partial nephrectomy could overestimate the incidence and severity of AKIs. In light of that, a recent study proposed new criteria for AKI after partial nephrectomy, which use an adjusted creatinine measurement as a postoperative baseline to determine AKI. Adjusted baseline creatinine was determined as the projection of the creatinine value after correcting it for the creatinine elevation due to the effect of parenchymal mass reduction [2]. There was a significant association between the renal functional recovery after surgery and AKI, determined by their proposed criteria, using adjusted baseline creatinine, but not for the AKIs determined by conventional criteria. However, another study using similar methodology reported no association between AKIs adjusted for parenchymal mass reduction and long-term renal function after partial nephrectomy [6].

**Figure 1:**
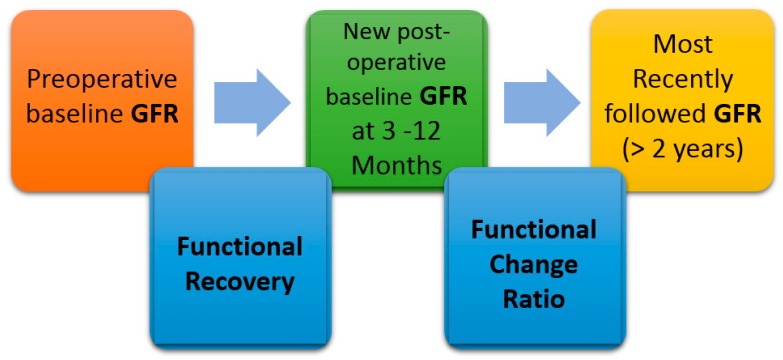
Study outcomes and time points of measurement. Functional recovery was measured as the ratio of glomerular filtration rate (GFR) at 3–12 months to preoperative baseline GFR. Functional change ratio was calculated as the ratio of most recent GFR to a new postoperative baseline GFR at 3–12 months. Preoperative baseline GFR was used as a baseline to calculate the outcome of functional recovery and a new post-operative baseline GFR at 3–12 months was used as a baseline to calculate the outcome of functional change ratio.

As such, the diagnosis of AKI and the influence of AKI on long-term renal function after partial nephrectomy, are still not clear. Therefore, we attempted to investigate the relationship between AKI after partial nephrectomy and long-term renal outcomes in our cohort. To evaluate AKI adjusted for the parenchymal mass reduction, we compared the association between AKI and postoperative long-term renal function by using both AKIs determined by unadjusted and adjusted baseline serum creatinine, accounting for parenchymal mass reduction. Previous studies investigated the long-term renal function after partial nephrectomy by measuring functional recovery and functional change ratio (FCR) (Figure 1) [2,6]. These outcomes measure the ratio of glomerular filtration rate (GFR) at different time points after surgery. We compared long-term renal function regarding two outcomes of functional recovery and FCR.

## 2. Materials and Methods

### 2.1. Study Population 

This single-center retrospective observational study was approved by the institutional review board of Seoul National University Hospital (1904-060-1026). Written informed consent was waived due to the retrospective nature of the present study. We reviewed electronic medical records of the patients who were ≥18 years old; had a renal mass and underwent partial nephrectomy, regardless of surgical techniques; and had a contralateral kidney between 2010 and 2014. Among the 639 patients who underwent partial nephrectomy, 629 patients were included in the final analysis, after excluding the patients without a preoperative, computed tomography (CT), coronal reconstruction image (*n* = 10).

### 2.2. Surgical and Anesthesia Procedure

Partial nephrectomies by open, laparoscopic and robot-assisted techniques were included in our analysis. Decisions regarding the type of surgical approach and use of warm versus cold ischemia were made based on the tumor characteristics. Surgical resection was performed after clamping the main renal artery or arteries. The renal vein was clamped selectively. Saline ice slush was used for cold ischemia. Anesthesia was induced and maintained by sevoflurane, desflurane or total intravenous anesthesia with propofol and remifentanil. All patients received an intraoperative 20 g mannitol infusion within 30 min before vascular clamping. For significant surgical bleeding, hydroxyethyl starch was administered to expand the intravascular volume and red blood cells were transfused to maintain the intraoperative hematocrit >24%.

### 2.3. Patient Data and Outcome Measurements

Demographic, baseline characteristics and surgery-related parameters that were known to be associated with renal function after nephrectomy were extracted from our electronic medical records (Table 1) [1,2,6,11,12,13]. Serum creatinine values measured 3 to 60 months after surgery were collected. GFR estimates were based on the equation in [14]. 

The primary outcome was a long-term FCR of GFR, which was defined as the most recent GFR/new baseline GFR after surgery [7]. New baseline GFR was defined as the latest value available during 3–12 months. Renal function is expected to recover a little after the sudden drop following partial nephrectomy, and the recovering GFR was defined as the new baseline GFR. This period of 3–12 months was chosen because individual follow-up duration varied and according to a previous study [6]. The most recent GFR collected was the GFR of at least 24 months after surgery. One secondary outcome was functional recovery from renal ischemia [2]. Functional recovery was calculated as the ratio of the percentage of function saved to the percent of parenchymal volume saved. The percentage of function saved was determined as the ratio of eGFR at 3–12 months after nephrectomy to the preoperative baseline eGFR. The summary and time points for these outcomes are shown in Figure 1. Outcome definitions and time points were selected to be the same as previous studies to compare results in the same time period [2,6].

**Figure 2:**
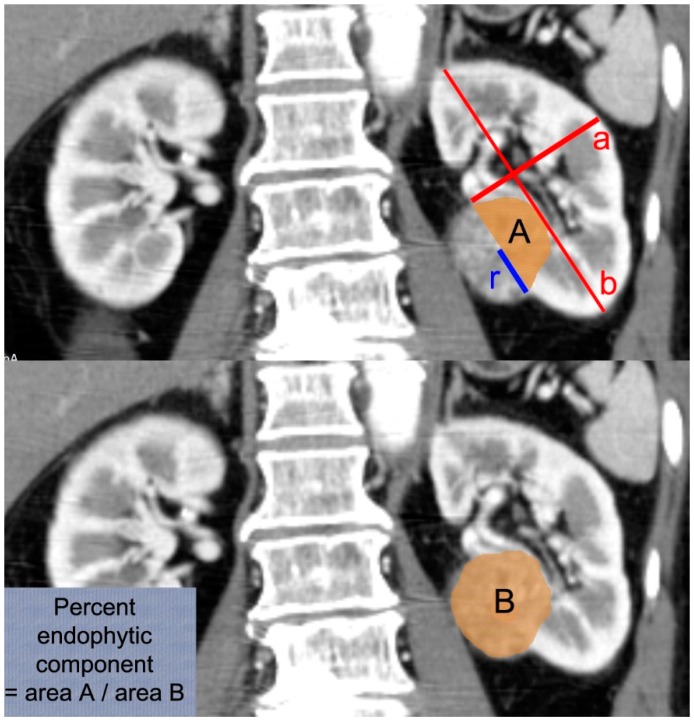
Measurements of the study. Kidney volume was estimated as cylindrical volume using the short (**a**) and long diameter (**b**) of the kidney on the three-dimensional computerized tomography (CT) image. The tumor volume was estimated as a ball with its radius of maximal tumor radius (r). Percent endophytic component was measured as a percentage ratio of endophytic tumor area (**A**) to whole tumor area (**B**) on the CT image where the maximal tumor area was observed.

Postoperative unadjusted AKI was defined by the creatinine criteria of KDIGO, which was determined according to the maximal change in serum creatinine level during the first seven postoperative days (stage 1, stage 2 and stage 3: 1.5–1.9, 2: 2–2.9 and a more than three-fold increase of preoperative baseline serum creatinine, respectively) [9,15]. The most recent serum creatinine level measured before surgery was used as the baseline. Adjusted AKI was defined by using the concept of previous studies [2,4], which set a new baseline adjusted creatinine after removing the contribution of parenchymal mass reduction and compared the postoperative peak serum creatinine level to the new baseline. We calculated the baseline adjusted creatinine by using the percentage of functional volume preservation (PFVP) based on the measurements on the preoperative CT image (Figure 2) [16,17]. The details of our calculation were described in Supplemental Text S1. By using the adjusted baseline, adjusted AKI was determined again using the same KDIGO criteria.

### 2.4. Statistical Analysis

SPSS software version 25.0 (IBM Corp., Armonk, NY, USA) and STATA/MP version 15.1 (StataCorp, College Station, TX, USA) were used to analyze the data. For all analyses, *p* < 0.05 was considered statistically significant. The Kolmogorov-Smirnov test was used to determine the normality of the data. All following analyses were performed separately for adjusted and unadjusted AKIs.

Firstly, baseline patient and surgical characteristics were compared between patients with and without adjusted AKIs. Mann–Whitney tests were used for continuous variables, and the chi-square test or Fisher’s exact test was used to compare incidence variables according to their expected counts. 

Secondly, we performed a multivariable linear regression analysis to elucidate significant predictors of the long-term FCR from the new baseline GFR after surgery to the most recent follow-up. Unadjusted and adjusted AKI stages were entered alternatively as a covariate to evaluate the association between AKI and FCR. Neither stepwise variable selection nor univariable screening was performed.

Thirdly, multivariable linear regression was performed to identify independent risk factors of the functional recovery during 3–12 months after partial nephrectomy. Unadjusted and adjusted AKI stages were considered the potential predictor alternatively.

Fourthly, univariable Spearman correlation analyses were performed to assess the relationships between the stages of adjusted AKIs and the longitudinal FCR. 

## 3. Results

Demographics and perioperative parameters are compared between adjusted AKI and no-AKI in Table 1 and between unadjusted AKI and no-AKI in supplemental Table S1. Baseline renal function is compared in supplemental Table S2. The incidence of unadjusted AKI was 16.5% (*n* = 104/629) (stage 1: *n* = 88 (14.0%); stage 2 or 3: *n* = 16 (2.5%)). The incidence of adjusted AKI was 8.6% (*n* = 54/629) (stage 1: *n* = 40 (6.4%); stage 2 or 3: *n* = 14 (2.2%)). Among all patients, only 19 patients (3.0%) required any renal replacement therapy during the postoperative hospital stay.

The median patient age was 54 years and the median parenchyma volume preservation was 89%. The median follow-up times for renal function were 27 and 28 months for patients with and without adjusted AKI (up to 61 months). The median FCR was 1.0 in patients without AKIs; and 0.92, 0.79 and 0.45 for patients with stage 1, 2 and 3 adjusted AKIs, respectively. The median functional recovery was 99% in patients without AKI; and 93%, 81% and 67% for patients with stage 1, 2 and 3 adjusted AKIs. There was no immediate postoperative mortality in our retrospective cohort. During the whole follow-up period, there were 17 (2.7%) cases of mortality from any cause and 13 (2.1%) cases of mortality from renal cell carcinoma.

**Figure 3:**
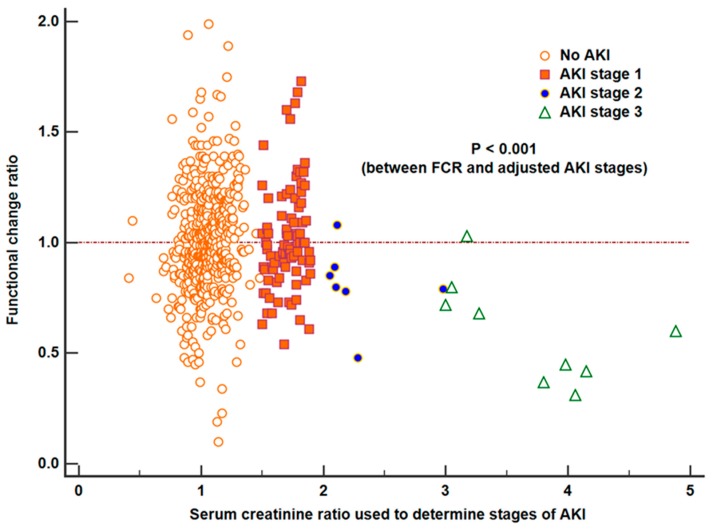
Distribution of long-term functional change ratio (FCR) across the different serum creatinine ratios used to determine acute kidney injury (AKI) stages. There was a significant correlation between FCR and adjusted AKI stages (*p* < 0.001).

Table 2 shows the results of multivariable linear regression analysis for FCR after partial nephrectomy. Postoperative adjusted AKI was identified as an independent risk factor for FCR of the most recent follow-up (*β* = −0.129 ± 0.026, *p* < 0.001), while unadjusted AKI was not. Performance of our multivariable prediction in terms of *R*^2^ was 0.10 and there was no significant multicollinearity between covariates. Figure 3 shows the distribution of FCR across the adjusted AKI stages. There was a significant correlation between FCR and adjusted AKI stages (*p* < 0.001, correlation coefficient −0.241).

As a sensitivity analysis, the association between functional recovery from ischemia at 3–12 months after partial nephrectomy was evaluated (Table 3). In the multivariable linear regression analysis, adjusted AKI stage was significantly associated with subsequent functional recovery (*β* = −0.243 ± 0.106, *p* = 0.023), while unadjusted AKI stage classified by the standard criteria failed to associate.

## 4. Discussion

We evaluated the association between AKI after partial nephrectomy, and long-term renal function. To account for the nephron mass reduction when diagnosing AKI, we determined AKI in two ways: using either unadjusted or adjusted baseline creatinine. Adjusted AKI after partial nephrectomy was a significant predictor of long-term FCR, but was not for unadjusted AKI. This significant association was consistent for both outcomes of FCR of at least two years after surgery and functional recovery of 3–12 months after partial nephrectomy.

AKI may be important for the long-term renal function only when it reflects true acute ischemic injury of the remaining renal nephrons. Efforts to reduce this ischemic injury may mitigate long-term renal dysfunction, thereby improving patient outcomes.

Partial nephrectomy has been regarded as standard therapy for patients with localized renal cancer [18]. Nevertheless, using the conventional diagnostic criteria of AKI, the incidence of AKI after partial nephrectomy was reported to be as high as 39%–54% [1,2]. Renal functional decline after partial nephrectomy is due to incomplete recovery of the remaining kidney from ischemic insult and parenchymal volume loss due to renal resection [4,19]. In surgeries other than nephrectomy, AKI is closely associated with the development of CKD and increased mortality [5,20,21]. However, for partial nephrectomy, this association has not been clearly ascertained. Previous studies reported varying results [2,6,7,8], possibly due to the difficulty there is to diagnose the pure renal parenchymal injury, and different study outcomes measuring long-term renal function at a varying intervals from nephrectomy.

Therefore, accurate diagnosis of AKI after partial nephrectomy is important, to evaluate the true impact of AKI on long-term renal function after nephrectomy. However, conventional AKI criteria simply compare the preoperative baseline creatinine level with postoperative values, not considering the aforementioned two components [2,9]. Although both of those two components could contribute to the long-term renal outcomes, our analysis indicated that the ischemic injury of the remaining kidney is more important for long-term renal prognosis. 

We estimated PFVP according to a previous method which measures the renal volume on the CT images using a cylinder volume ratio method [16]. Although this is a simple and easy estimation of kidney volume, this could be inaccurate. We calculated adjusted baseline serum creatinine and eGFR by using this estimated PFVP under the assumption that endophytic components of the kidney lose functioning nephrons due to surgical resection. However, previous studies of partial nephrectomy used volumetric analysis using pre and postoperative CT images, which could be more accurate [2,6,22]. The spherical cap surface model used only the preoperative CT image to measure PFVP like our study [17]. However, they calculated the volume of normal parenchyma removed during surgery, which was not considered in our calculation. Although not available in our data, the surgeon’s visual assessment of parenchymal preserved volume was reported to be strongly associated with measured values [23]. Preoperative assessment of PFVP based on preoperative imaging provided similar predictive capacity to the surgeon’s assessment [24]. Our study results should be interpreted under these limitations. 

There have been two studies investigating the association between AKI and long-term renal function after partial nephrectomy [2,6]. Both studies used a volumetric analysis of pre and postoperative CT images. Zhang et al. defined adjusted AKI according to projected serum creatinine as a new baseline and reported a significant association between AKI using a creatinine-adjusted baseline, and early renal functional recovery after partial nephrectomy [2]. This study used functional recovery at 3–12 months after partial nephrectomy and the preoperative baseline. The median parenchymal mass reduction was 11% and cold ischemia was used 53% of the time. However, in another study, which evaluated FCR at least after 12 months after partial nephrectomy compared to the postoperative, new baseline at 3–12 months after surgery, reported no significant association between adjusted AKI stages and FCR [6]. The median parenchymal mass reduction was 20% and cold ischemia was used 47% of the time. Although adjusted AKI was used in both studies, varying follow-up duration and different baseline of renal function could yield different results. Different sizes of the tumors, degrees of parenchymal resection, the incidence of cold ischemia, ischemic time and tumor complexity, could influence long-term renal function after partial nephrectomy. A patient cohort with shorter ischemic time and higher incidence of cold ischemia may lead to relatively good long-term renal function, and a population with a longer ischemic time and low incidence of cold ischemia may yield poor long-term renal function, which could discriminate the influence of AKI. We used the same outcomes as those two studies for a valid comparison.

For the patients undergoing unilateral radical nephrectomy, there have been only two studies, which reported a significant association between postoperative AKI and progressive CKD [7,8]. The incidence of AKI was rather high, up to 49.1%, and the association was strong, with a three to four-fold higher risk, although CKD was defined differently between the two studies. Further prospective studies are required to validate this association. 

Although functional recovery after partial nephrectomy is mainly determined by parenchymal volume preservation [22], we demonstrated that ischemic insult of the remaining kidney could also affect the functional outcome substantially if ischemic time is prolonged. Efforts have been made to reduce renal injury after partial nephrectomy. The effects of pharmacological agents, such as mannitol and dopamine, have been questioned [25,26]. Cold ischemia is known to be effective in restoring renal function after partial nephrectomy [27]. However, cold ischemia was not significant in the present study, possibly due to its low incidence in our cohort. Recently, zero ischemia partial nephrectomy or selective arterial clamping has been suggested [28,29]. Remote ischemic conditioning using transient limb ischemia was suggested to reduce short-term renal functional impairment after a laparoscopic partial nephrectomy [30,31]. A previous pilot study reported combined treatment of ketorolac and remote ischemic conditioning in patients undergoing partial nephrectomy reduced the incidence of AKI [32]. Hydrogen sulfide was effective in attenuating prolonged warm renal ischemia-reperfusion injury in a previous animal study [33]. However, the incidence of AKI and CKD after partial nephrectomy was still high. Further studies for these interventions may consider the outcome of AKI based on adjusted creatinine. 

Most of the significant predictors for new-onset CKD are consistent with previous studies. Although unmodifiable, preoperative proteinuria is a known predictor [1,11,34], which was consistent with our results. Renal ischemic time [1,34,35] should be reduced and cold ischemia can be applied during renal mass resection to reduce renal injury, despite existing controversy [4,36,37]. 

This study has several limitations. Firstly, our study was a retrospective study of a single tertiary care center. Unknown and unmeasured biases could have affected our results. Open and minimally invasive surgeries are mixed in our population. Missing values of long-term renal function jeopardize the validity of our results. Secondly, as mentioned earlier, our estimation of adjusted baseline creatinine or eGFR has limitations. Further studies are required to support our results with validated volumetric analysis. Thirdly, we included patients who have bilateral kidneys in our analysis. Although all patients underwent partial nephrectomy, the adjustment of baseline creatinine could be more inaccurate in a patient with bilateral kidneys compared to one with a single kidney.

## 5. Conclusions

By using baseline creatinine corrected for parenchymal mass reduction based on our simple measurements on preoperative CT, AKI adjusted for the renal parenchymal mass reduction was a significant predictor of long-term renal function for at least two years after surgery, while unadjusted AKI was not. We demonstrated this association by using two different outcomes that previous studies used. Our study suggests the prognostic implication of acute injury of remaining renal parenchyma during partial nephrectomy, in regard to long-term renal function. Efforts to reduce the remaining renal parenchymal injury may contribute to mitigate the risk of long-term deterioration in renal function after partial nephrectomy. However, prospective trials with validated renal volume measurements are required.

## Figures and Tables

**Table 1:** Patient characteristics and perioperative parameters according to acute kidney injury adjusted for parenchymal mass reduction.

Characteristic	Adjusted AKI	No AKI	*p*-Value
Patient population, *n*	54 (8.6)	575 (91.4)	
Demographic data			
Age, yr	61 (51–67)	54 (46–65)	0.020
Female, *n*	3 (5.6)	176 (30.6)	<0.001
Body-mass index, kg/m^2^	24.2 (22.5–26.7)	24.6 (22.6–26.7)	0.599
Background medical status			
Hypertension, *n*	27 (50.0)	202 (35.1)	0.030
Diabetes mellitus, *n*	9 (16.7)	75 (13.0)	0.409
Cerebrovascular accident, *n*	1 (1.9)	14 (2.4)	0.781
Angina pectoris, *n*	2 (3.7)	4 (0.7)	0.087
Preoperative hemoglobin, g/dL	14.0 (11.1–15.2)	14.1 (12.9–15.0)	0.147
Preoperative serum albumin level, g/dL	4.3 (3.9–4.5)	4.5 (4.2–4.6)	0.001
Preoperative proteinuria, *n*	8 (14.8)	27 (4.7)	0.007
Unilateral kidney, *n*	8 (14.8)	51 (8.9)	0.147
Operation and anesthesia details			
Surgery type, *n*			0.965
Laparoscopic	1 (1.9)	31 (5.4)	
Robot-assisted	6 (11.1)	122 (21.2)	
Open	47 (87.0)	422 (73.4)	
Clinical stage, *n*			<0.001
T1a/T1b	35 (64.8)/12 (22.2)	496 (86.3)/62 (10.8)	
T2a/T2b	3 (5.6)/2 (3.7)	14 (2.4)/2 (0.3)	
T3a/T3b/T3c	1 (1.9)/1 (1.9)/0	1 (0.2)/0/0	
N 0/1	51 (94.4)/3 (5.6)	571 (99.3)/4 (0.7)	0.016
M 0/1	51 (94.4)/3 (5.6)	567 (98.6)/8 (1.4)	0.060
R.E.N.A.L. score	7 (7–8)	6 (5–7)	<0.001
Low (4–6)	9 (16.7)	390 (67.8)	
Intermediate (7–9)	42 (77.8)	179 (31.1)	
High (10–12)	3 (5.6)	6 (1.0)	
Tumor maximal diameter, cm	2.5 (2.0–4.0)	2.3 (1.5–3.5)	0.038
Operation time, min	150 (120–206)	140 (107–180)	0.113
Warm ischemia, *n*	51 (94.4)	548 (95.3)	0.736
Cold ischemia, *n*	3 (5.6)	27 (4.7)	0.736
Renal ischemic time, min	30 (24–42)	24 (17–30)	<0.001
Warm ischemic time, min	28 (24–42)	24 (18–30)	<0.001
Cold ischemic time, min	38 (30–38)	31 (17–39)	0.387
Estimated parenchymal volume preservation, %	89 (85–90)	89 (88–90)	0.623
Anesthesia technique			0.113
Total intravenous agent, *n*	50 (92.6)	483 (84.0)	
Inhalational agent, *n*	4 (7.4)	92 (16.0)	
Intraoperative vasopressor use, *n*	7 (16.3)	66 (12.0)	0.467
Bleeding and transfusion amount			
pRBC transfusion, *n*	10 (18.5)	20 (3.5)	<0.001
Estimated blood loss, mL	300 (200–600)	200 (100–350)	<0.001
Input and output during surgery			
Crystalloid administration, mL	1150 (800–1700)	1200 (800–1700)	0.653
Colloid administration, mL	0 (0–500)	0 (0–400)	0.486

Data are presented as median (interquartile range) or number (%). AKI = acute kidney injury; R.E.N.A.L. = radius, exophytic/endophytic properties, nearness of tumor to collecting system or sinus, anterior/posterior, hilar, location relative to polar lines; pRBC = packed red blood cell.

**Table 2:** Multivariable linear regression analysis of functional change ratio after partial nephrectomy.

Variable	*β* ± Standard Error	*p*-Value	VIF
Age, per 10 yr	0.003 ± 0.009	0.741	1.48
Male	−0.014 ± 0.024	0.556	1.34
Body-mass index, kg/m^2^	0.008 ± 0.003	0.107	1.16
Hypertension	−0.011 ± 0.022	0.622	1.28
Diabetes mellitus	0.016 ± 0.030	0.608	1.14
Preoperative hemoglobin concentration, g/dL	0.014 ± 0.007	0.051	1.56
Preoperative albumin level, g/dL	0.065 ± 0.026	0.011	1.41
Preoperative proteinuria	−0.147 ± 0.054	0.007	1.23
Preoperative estimated glomerular filtration rate, mL/min/1.73 m^2^	0.001 ± 0.001	0.160	1.13
Surgery type, open versus minimal invasive surgery	−0.014 ± 0.023	0.555	1.20
Renal ischemia time, per 10 min	−0.026 ± 0.009	0.008	1.21
Ischemia type (cold)	0.036 ± 0.047	0.444	1.06
Maximal diameter of renal mass, cm	−0.005 ± 0.007	0.410	1.16
Adjusted acute kidney injury grade *	−0.129 ± 0.026	<0.001	1.10
OR unadjusted acute kidney injury grade	−0.011 ± 0.020	0.573	1.32

VIF = variance inflation factor. * Adjusted acute kidney injury was determined based on adjusted baseline creatinine value accounting for parenchymal mass reduction. Functional change ratio was determined as the ratio of most recent glomerular filtration rate (GFR) (at least 24 months after surgery) to GFR at 3 to 12 months.

**Table 3:** Multivariable linear regression analysis of functional recovery from ischemia at 3–12 months after partial nephrectomy.

Variable	*β* ± Standard Error	*p*-Value	VIF
Age, per 10 yr	−0.019 ± 0.049	0.706	1.48
Male	−0.186 ± 0.130	0.152	1.31
Body-mass index, kg/m^2^	−0.024 ± 0.017	0.162	1.17
Hypertension	−0.027 ± 0.122	0.826	1.27
Diabetes mellitus	−0.073 ± 0.166	0.663	1.14
Preoperative hemoglobin concentration, g/dL	0.020 ± 0.041	0.631	1.55
Preoperative albumin level, g/dL	0.130 ± 0.136	0.338	1.29
Preoperative estimated glomerular filtration rate, mL/min/1.73 m^2^	0.001 ± 0.003	0.726	1.25
Renal ischemia time, per 10 min	−0.057 ± 0.048	0.236	1.17
Ischemia type (cold)	0.774 ± 0.262	0.003	1.06
Maximal diameter of renal mass, cm	−0.230 ± 0.037	<0.001	1.23
Adjusted acute kidney injury stage *	−0.243 ± 0.106	0.023	1.28
OR unadjusted acute kidney injury stage	−0.177 ± 0.118	0.137	1.15

VIF = variance inflation factor. Functional recovery was calculated as the ratio of the percentage of function saved to the percentage of parenchymal volume saved. * Adjusted acute kidney injury was determined based on adjusted baseline creatinine value accounting for parenchymal mass reduction. Percent function saved was determined as the ratio of estimated glomerular filtration rate (eGFR) at 3–12 months after nephrectomy to preoperative baseline eGFR.

## Supplementary Materials

The following are available online at https://www.mdpi.com/2077-0383/8/9/1482/s1, Supplemental Text S1. Calculation of adjusted preoperative estimated glomerular filtration rate and adjusted serum creatinine in partial nephrectomy. Supplemental Table S1. Patient characteristics and perioperative parameters according to acute kidney injury using unadjusted preoperative baseline creatinine. Supplemental Table S2. Comparison of baseline renal function and the rates of surgical complications between those with and without adjusted acute kidney injury.
