# Supplementary material for: Acute Kidney Injury Adjusted for Parenchymal Mass Reduction and Long-Term Renal Function after Partial Nephrectomy
Source: J Clin Med. 2019 Sep 18;8(9):1482. doi: 10.3390/jcm8091482 (PMC6780324; doi:10.3390/jcm8091482)
Supplement: Supplementary file 1 [file jcm-08-01482-s001.pdf]

Acute Kidney Injury Adjusted for Parenchymal Mass Reduction and Long-Term Renal Function after Partial Nephrectomy

Running title: Acute kidney injury after partial nephrectomy

Hyun-Kyu Yoon, MD[1], **Ho-Jin Lee**, MD[1], **Seokha Yoo**, MD[1], **Sun-Kyung Park**, MD[1], **Yongsuk Kwon**, MD[1], **Kwanghoon Jun**, MD[1], **Chang Wook Jeong**, MD,PhD[2], **Won Ho Kim**, MD,PhD[1*]

[1]Department of Anesthesiology and Pain Medicine, and [2]Department of Urology, Seoul National University Hospital, Seoul National University College of Medicine, Seoul, Republic of Korea

Item	Title	Page
**Supplemental Text S1**.	Calculation of adjusted preoperative estimated glomerular filtration rate (eGFR) and adjusted serum creatinine in partial nephrectomy	2
**Supplemental Table S1**.	Patient characteristics and perioperative parameters according to acute kidney injury using unadjusted preoperative baseline creatinine	5
**Supplemental Table S2.**	Comparison of baseline renal function and the rates of surgical complications between those with and without adjusted acute kidney injury.	7

Supplemental Text S1. Calculation of adjusted preoperative estimated glomerular filtration rate (eGFR) and adjusted serum creatinine in partial nephrectomy

First, we measured the short and long diameter of kidney on the coronal computerized tomography (CT) image as follows.

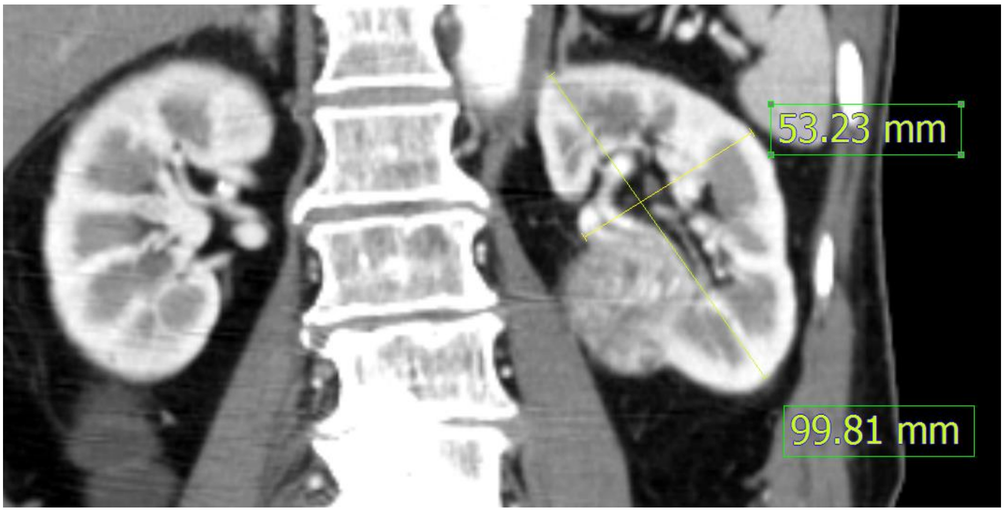

Then, we estimated the cylindrical volume of kidney by the following equation.

$$\text{Vkid (Kidney volume)} = \pi \times \left(\frac{diameter\ of\ short\ axis}{2}\right)^2 \times (diameter\ of\ long\ axis)$$

Then, we measured the volume of tumor assuming that the mass is a ball with its radius of maximal tumor radius.

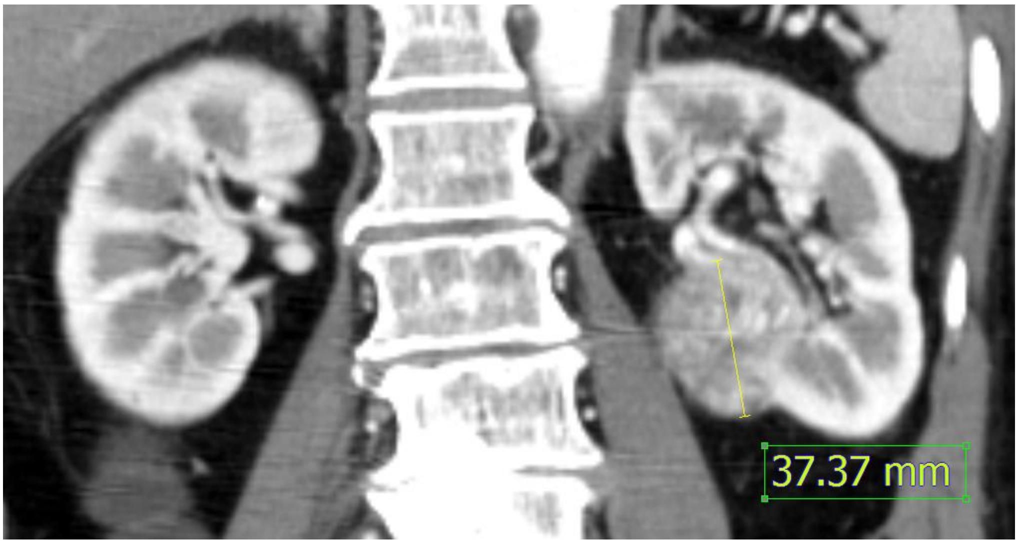

$$\text{Vtum (tumor volume)} = \frac{4}{3} \times \pi \times (maximal\ tumor\ radius)^3$$

$$\text{Vnfv (non} - \text{functional kidney volume)} = Vtum \times (\%\ endophytic\ component)$$

Percent endophytic component was measured on the CT image where the maximal radius of tumor is found as the following figure. Percent endophytic component was measured as the ratio of endophytic tumor area (B) to whole tumor area (A).

$$\%\ \text{endophytic component} = \frac{B \times 100}{A}$$

Each area was measured using the planimetry function of the picture archiving and communication system (PACS) of our institution.

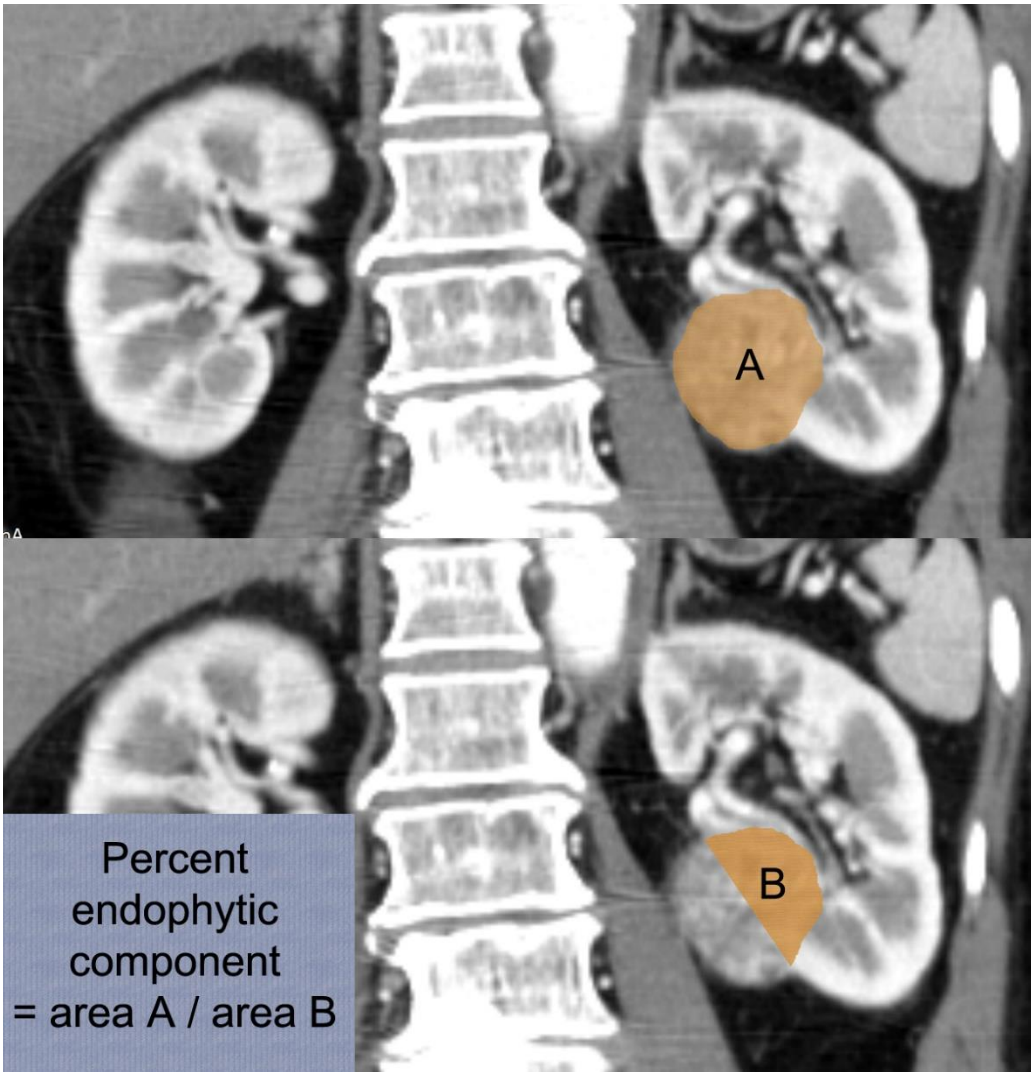

$$\text{Adjusted preoperative renal volume (V}'\text{kid)} = Vkid - Vnfv$$

$$\text{PFVP (Percent functional volume preservation)} = \frac{V'kid}{Vkid} = \frac{Vkid - Vnfv}{Vkid}$$

$$\text{Adjusted preoperative baseline estimated glomerular filtration rate (eGFR)}$$

$$= (eGFR\ of\ the\ non\_operating\ kidney$$

$$+ (eGFR\ of\ the\ operating\ kidney)$$

$$= \frac{1}{2} \times (Preoperative\ eGFR) + \frac{1}{2} \times (Preoperative\ eGFR) \times PFVP$$

We calculated adjusted preoperative baseline serum creatinine from the Modification of Diet in Renal Disease (MDRD) equation with known eGFR and unknown creatinine as follows.

$$\text{eGFR} = 186 \times (serum\ creatinine)^{-1.154} \times age^{-0.203} \times 0.742\ (for\ female)$$

$$\text{Log (eGFR)} = Log\ 186 - 1.154 \times Log\ (serum\ creatinine) - 0.203 \times Log\ (age) + Log\ 0.742\ (for\ female)$$

$$1.154 \times \text{Log (serum creatinine)} = 5.2257 - Log\ (eGFR) - 0.203 \times Log\ (age) - 0.2984\ (for\ female)$$

$$\text{Log (serum creatinine)} = \frac{5.2257 - \log(eGFR) - 0.203 \times Log(age) - 0.2984\ (for\ female)}{1.154}$$

Finally,

$$Adjsuted\ serum\ creatinine = e^{\frac{5.2257 - \log eGFR - \log age - 0.2984\ (for\ female)}{1.154}}$$

When the tumor margin inside kidney is irregular, it was difficult to delineate the endophytic portion of the tumor accurately. For these cases, measurement was repeated three different times and average value was used.

Supplemental Table S1. Patient characteristics and perioperative parameters according to acute kidney injury using unadjusted preoperative baseline creatinine

Characteristic	Unadjusted AKI	No AKI	*P*-value
Patient population, n	104 (16.5)	525 (83.5)	
Demographic data			
Age, yr	57 (45 – 65)	55 (47 – 65)	0.731
Female, n	12 (11.5)	167 (31.8)	<0.001
Body-mass index, kg/m^2	24.5 (22.8 – 26.8)	24.6 (22.5 – 26.8)	0.501
Background medical status			
Hypertension, n	44 (42.3)	185 (35.2)	0.171
Diabetes mellitus, n	18 (17.3)	66 (12.6)	0.195
Cerebrovascular accident, n	5 (4.8)	10 (1.9)	0.085
Angina pectoris, n	4 (3.8)	2 (0.4)	0.008
Preoperative hemoglobin, g/dl	14.0 (12.3 – 15.2)	14.1 (12.9 – 14.9)	0.892
Preoperative serum albumin level, mg/dl	4.3 (4.1 – 4.6)	4.5 (4.2 – 4.6)	0.037
Preoperative proteinuria, n	10 (9.6)	25 (4.8)	0.060
Preoperative unadjusted serum creatinine, mg/dl	0.96 (0.85 – 1.12)	0.90 (0.78 – 1.03)	<0.001
Adjusted serum creatinine, mg/dl	1.07 (0.94 – 1.25)	1.01 (0.86 – 1.16)	<0.001
Preoperative unadjusted GFR, calculated by MDRD, ml/min/1.73 m^2	81 (65 – 94)	82 (71 – 94)	0.240
Preoperative adjusted GFR, ml/min/1.73 m^2	73 (54 – 89)	78 (65 – 91)	0.032
Unilateral kidney, n	13 (12.5)	46 (8.8)	0.232
Operation and anesthesia details			
Preoperative unadjusted, n			0.071
GFR ≥ 90 ml/min/1.73 m^2	31 (4.9)	173 (27.5)	
60 ≤ GFR < 89 ml/min/1.73 m^2	61 (58.7)	331 (63.0)	
GFR < 60 ml/min/1.72 m2	12 (11.5)	21 (4.0)	
Surgery type, n			0.865
Laparoscopic	4 (3.8)	28 (5.3)	
Robot-assisted	84 (80.8)	385 (73.3)	
Open	16 (12.5)	112 (21.3)	
Clinical stage, n			<0.001
T1a/ T1b	73 (70.2)/ 22 (21.2)	458 (87.2)/ 52 (9.9)	
T2a/ T2b	5 (4.8)/ 2 (1.9)	12 (2.3)/ 2 (0.4)	
T3a/ T3b / T3c	1 (1.0)/ 1 (1.0)	1 (0.2)/ 0	
N 0/1	101 (97.1)/ 3 (2.9)	521 (99.2)/ 4 (0.8)	0.059
M 0/1	99 (95.2)/ 5 (4.8)	519 (98.9)/ 6 (1.1)	0.023
R.E.N.A.L. score	7 (7 – 8)	6 (5 – 7)	<0.001
Tumor maximal diameter, cm	2.6 (2.1 – 3.8)	2.3 (1.5 – 3.5)	0.005
Operation time, min	155 (120 – 210)	140 (105 – 180)	0.003
Parenchymal mass preservation, %	89 (85 – 90)	89 (88 – 90)	0.762

Anesthesia technique			0.040
Total intravenous agent, n	95 (91.3)	438 (83.4)	
Inhalational agent, n	9 (8.7)	87 (16.6)	
Renal ischemic time, min	29 (24 – 40)	23 (17 – 30)	<0.001
Cold ischemia, n	8 (7.7)	22 (4.2)	0.132
Intraoperative vasopressor use, n	17 (18.5)	56 (11.2)	0.052
Bleeding and transfusion amount			
pRBC transfusion, n	15 (14.4)	15 (2.9)	<0.001
Estimated blood loss, ml	250 (150 – 550)	200 (100 – 300)	<0.001
Input and output during surgery			
Crystalloid administration, ml	1400 (900 – 1900)	1100 (800 – 1700)	0.003
Colloid administration, ml	0 (0 – 400)	0 (0 – 400)	0.779

Data are presented as median (IQR) or number (%).

AKI = acute kidney injury; IQR = interquartile range; pRBC = packed red blood cell.

Supplemental Table S2. Comparison of baseline renal function and the rates of surgical complications between those with and without adjusted acute kidney injury.

Cutoff	Adjusted AKI (n = 54)	No AKI (n = 575)	p value
Serum creatinine, mg/dl			
Preoperative unadjusted	1.01 (0.87 – 1.20)	0.90 (0.78 – 1.03)	<0.001
Adjusted, based on parenchymal mass reduction	1.12 (0.96 – 1.33)	1.01 (0.87 – 1.20)	<0.001
eGFR, calculated by MDRD, ml/min/m^2			
Preoperative, unadjusted	74 (62 – 89)	82 (71 – 94)	0.003
Preoperative adjusted	67 (46 – 83)	78 (65 – 91)	<0.001
Preoperative unadjusted, n			0.001
GFR ≥ 90 ml/min/1.73 m^2	13 (24.1)	191 (33.2)	
60 ≤ GFR < 89 ml/min/1.73 m^2	30 (55.6)	362 (63.0)	
GFR < 60 ml/min/1.72 m2	11 (20.4)	22 (3.8)	
Postoperative complications by Clavien-Dindo classification, n			0.172
None	49 (90.7)	550 (95.7)	
Grade 1	1 (1.9)	7 (1.2)	
Grade 2	2 (3.7)	3 (0.5)	
Grade 3a	0	5 (0.9)	
Grade 3b	2 (3.7)	10 (1.7)	

AKI = acute kidney injury; IQR = interquartile range. eGFR = estimated glomerular filtration rate; MDRD = modification of diet and renal disease; CKD = chronic kidney disease;
